# Dr. Edward Maynard

**Published:** 1891-06

**Authors:** 


### Obituary.

### DR. EDWARD MAYNARD.

Dr. Edward Maynard died May 4, 1891, after a long and painful illness. He was born in Madison, N. Y., April 26, 1813, and consequently was aged seventy-eight years at the time of his death. Dr. Maynard was twice married. By the first marriage, with Miss Ellen Sophia Doty, of Sherburne, N. Y., he had six children, all of whom are living,—Mr. George W. Maynard, New York; Dr. John D. Maynard, dentist, New York; Mrs. J. L. Hatch; Mrs. D. J. Hatch, of Rochester; and the Misses Marie and Virginia Maynard, of Washington, D. C.

The second marriage was to Miss Nellie Long, of Savannah, Ga. She, with the only child of this union, Mrs. Edwin Q. Laselle, of Troy, N. Y., survive him.

Dr. Maynard was prepared at Hamilton Academy for the Military Academy at West Point, entering that institution in 1831. The drill duty there being too exacting for his always delicate health, he resigned in the same year, and gave his attention to civil engineering, the study of anatomy, architecture, drawing, and such mechanical employment as would best educate his hands for the profession he finally adopted in 1835. He settled in Washington, D. C., in the following year, and continued to practise there, with short intervals, up to March, 1890. His inventions in instruments and modes of using them in his profession have been numerous, and many of them have become well known and generally adopted. His discovery of the very great diversity of situation, form, and capacity of the maxillary antra was made known to the Faculty of the Baltimore College of Dental Surgery in 1846, and was officially acknowledged. It has from that time been regarded as of great importance in the treatment of disease of the superior maxillaries.

His announcement of the existence of dental fibrils, based upon his discovery that sensitive dentine could be cut with less pain in particular directions than in opposite ones, was discussed, and the discussion was reported in the *Transactions* of the American Society of Dental Surgeons before any announcement of the discovery of such fibrils by the aid of the microscope.

So far as now known he was the first to successfully practise (in 1838) the thorough filling, with gold-foil, of the nerve-cavity, including the nerve-canals, in molar and bicuspid teeth, an operation he introduced in Europe in 1845. At St. Petersburg, the emperor's physician, Dr. Arndt, having witnessed this operation, Dr. Maynard was immediately employed in the imperial family as court dentist.

The emperor (Nicolas I.) offered to create the title of "Actual Dentist to His Imperial Majesty," with the rank of major, and give him this if he would remain in Russia for ten years and practise and teach his ways of practice; Dr. Maynard to be attached to the court with a salary or have a private practice as he might elect. This offer was declined. Accompanying the sum paid for his services was a magnificent diamond ring, sent as a token of appreciation.

In 1857 he accepted the chair of Theory and Practice in the Baltimore College of Dental Surgery (the first dental college ever established), and held the like position in the Faculty of the Dental Department of the National University at Washington. His practice has been mainly among the higher classes, including several Presidents, a great number of Cabinet officers, senators, representatives, officers of the army and navy, foreign ministers, and others, who demand such dental operations as necessarily exclude all machine-work.

He has received the honorary degrees of A.M., M.D., and D.D.S., is an honorary member of the American Academy of Dental Science of the European Society of American Dentists, member of International Medical Congress, September, 1887, etc.

In 1845 (March 3) Dr. Maynard patented a system of priming for fire-arms, to take the place of the percussion-cap. Coiled and protected in a recess of the lock was a water-proof, incombustible, tape-like paper strip, having on one side fifty lozenge-like elevations at equal distances apart.

Each elevation contained a charge of fulminate. When the hammer was cocked, one charge was automatically projected over the nipple. When the hammer descended, it cut off and fired the charge. The United States government bought the right to use this invention, and, after many years of delay, applied it to about thirty thousand rifles and muskets. It was honored abroad also, the King of Belgium complimenting the inventor in person as the "author of such a beautiful invention," and offering to apply it on trial to the muskets of a regiment of his soldiers, the King of

Prussia making him a chevalier of the Military Order of the Red Page, and the King of Sweden giving him the great medal of merit,—an honor rarely given to a foreigner.

In 1851 (May 27, 1859, December 6, second patent) he patented a breech-loading rifle, known now throughout and beyond the limits of civilization as the Maynard rifle. His later improvements on the mechanism of this arm (those patented in 1859) adapted the arm to the use of his invention in metallic ammunition (patented 1856, June 17), in which a truncated cylindro-conoidal projectile is tightly set in a cylindrical metallic cartridge, having and holding firmly the axis of the projectile in the axis of the cartridge: thus, in the act of loading a gun, placing the axis of the projectile precisely in the line of the axis of the bore of the gun and holding it in that line until it, in the act of firing, has fully entered the bore. As the use of this kind of ammunition resulted in great increase in precision, and on frontier trial proved its ability to withstand all the casualties of rough service, it eventually came to be adopted by the United States government, by all American manufacturers of breech-loading arms, and is now in use by nearly all nations for their military rifles, and by nearly all riflemen in all countries.

In 1860 (October 30) he patented a method of converting muzzle-loading arms into breech-loaders. The Secretary of War ordered the two master armorers, Allin, of Springfield Armory, and Ball, of Harper's Ferry Armory, to examine and report at Washington upon this invention. They made a detailed estimate of the cost of converting muskets and of making new ones on this system, and reported that they had tried it and found it safe, strong, and durable. The principal claim in this patent is on a device, then first applied, of relieving the hinge of the recoil-block from strain by compelling all the rearward pressure to come against the breech-pin or other solid rear end of the barrel. Under various modifications of form and position this particular feature has come into extensive use in military arms in the United States and other countries.

In 1868 (October 20) he patented the joining together of two rifle or shot barrels by a device that would allow either barrel to expand or contract endwise, independently of the other. This proved especially valuable in double rifles, inasmuch as by the old method of joining the barrels immovably, either barrel may be so heated by a single shot, or by having the sun shine on it while the other is in shade, that it will become measurably longer than the one not so heated, resulting in both barrels being made crooked and kept so until the heat is equalized by convection.

In 1886 (June 8) he patented an invention for indicating the number of cartridges in the magazine of a repeating fire-arm at any time. A disk of metal, say a quarter of an inch thick and of the diameter of the magazine, forms the front end of it. This disk has figures plainly marked on its circumference from 0 to the full number the magazine can contain. It is connected with the magazine spring, which revolves in one direction as cartridges are put into the magazine and in the opposite direction when they are pushed out. The value of the invention is readily appreciated by military officers, to whom it is of prime importance that they should be able to tell at a glance to what extent, if at all, the magazine is supplied before going into action, and by hunters for a similar reason.

Many other patents were granted to Dr. Maynard, many of them subsidiary, on fire-arms and ammunition. None of these are deemed to be of sufficient note by themselves for mention herein.

